# Quality Improvement Initiative in a Community Hospital to Reduce Central Line Device Utilization Rate

**DOI:** 10.7759/cureus.41037

**Published:** 2023-06-27

**Authors:** Esraa Hassan, Bijoy Mathew, Jessica Poehler, Kimberly Kopischke, Greta Zoesch, Noura Attallah, Abbas B Jama, Nitesh K Jain, Eric O Gomez Urena, Syed Anjum Khan

**Affiliations:** 1 Critical Care Medicine, Mayo Clinic Health System, Mankato, USA; 2 Strategy Consulting Services, Mayo Clinic, Rochester, USA

**Keywords:** central line-associated bloodstream infection (clabsi), intensive care unit, device utilization rate, quality improvement, central line, dmaic, icu, central line utilization

## Abstract

Background

The intensive care unit (ICU) in a community hospital in southwest Minnesota saw a steady increase in central line-associated bloodstream infections (CLABSI) and an increase in the utilization of central lines. The baseline CLABSI rate was 11.36 at the start of the project, which was the highest in the last five years. The corresponding device utilization rate (DUR) was 64%, which increased from a pre-COVID pandemic rate of 45%.

Aim

The aim of this project was to decrease the ICU DUR by 37.5% from a baseline of 64% to 40% within six months without adversely impacting staff satisfaction.

Methods

A multidisciplinary team using the define, measure, analyze, improve, and control (DMAIC) methodology reviewed the potential causes of the increased use of central lines in the ICU. The team identified the following major causal themes: process, communication, education, and closed-loop feedback. Once the root causes were determined, suitable countermeasures were identified and implemented to address these barriers. These included reviewing current guidelines, enhanced care team rounding, staff education, and the creation of a vascular access indication algorithm. The team met biweekly to study the current state, determine the future state, evaluate feedback, and guide implementation.

Results

The pandemic saw a surge in the number of severely ill patients in the ICU, which may have caused an increase in the DUR. The project heightened the awareness of the increased DUR and its impact on the CLABSI rate. The initiation of discussion around this project led to an immediate decline in DUR via increased awareness and focus. As interventions were introduced and implemented, the DUR continued to decrease at a steady rate. Post implementation, the DUR met the project goal of less than 40%. The team continued to track progress and monitor feedback. The DUR continued to meet the goal for three months post implementation. Since the start of the project, there have been no CLABSI events reported. This effort has positively impacted safety and patient outcomes.

Conclusions

Through a defined process, the central line utilization rate in our ICU was decreased to 37.5% to meet the target goal and has been sustained.

## Introduction

Reducing the use of central lines can decrease complications in intensive care unit (ICU) patients [1]. Most patients admitted to an ICU undergo intravenous cannulation. Peripheral venous cannulation usually is attempted first, as peripheral veins are readily accessible. Also, large-bore, relatively short catheters facilitate rapid fluid infusion, which is commonly used during initial resuscitation efforts [2]. More than five million central venous catheterizations (CVCs) are performed annually in the United States [3]. More than 15% of patients who undergo this procedure experience one or more complications. Some of the complications include pneumonia, subcutaneous hematoma, hemothorax, cardiac arrest, arterial puncture, catheter malposition, and inability to insert the catheter. Although coagulopathy increases the risk of hemorrhage following CVC, bleeding problems can be minimized with cautious site selection and attentive procedure. There are no definite contraindications because CVC may sometimes be lifesaving for patients [4,5]. When microbes enter the bloodstream through the central line, a severe infection known as central line-associated bloodstream infection (CLABSI) may develop [6]. Increased morbidity, mortality, and medical expenses are linked to CLABSI [6]. The National Healthcare Safety Network (NHSN) of the Centers for Disease Control and Prevention (CDC) defines CLABSI as a primary bloodstream infection (BSI) in a patient who had a central vascular catheter within the previous 48 hours, and it is not a bloodstream infection related to an infection at another site [7]. For the pathological diagnosis of catheter-related bloodstream infection (CRBSI), positive blood cultures are required in addition to microbiological data establishing the catheter as the source of the bloodstream infection [7].

## Materials and methods

**Figure 1:**
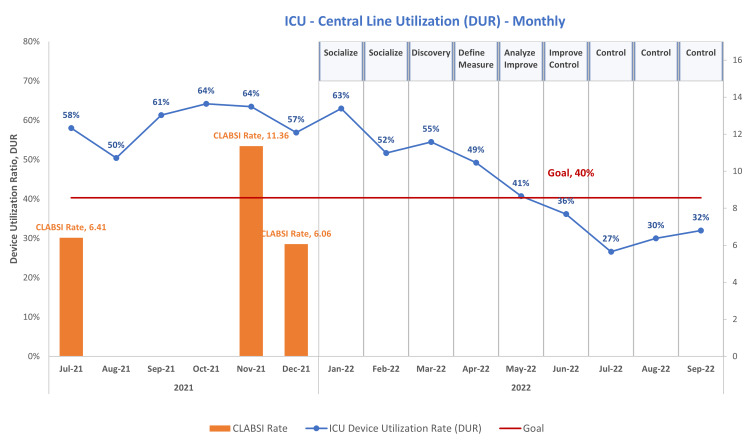
Device utilization rate (DUR) Notes: CLABSI rate is the number of CLABSI cases / central line days × 1000​. Device utilization rate (DUR) is the central line days / patient days​ CLABSI, central line-associated bloodstream infections; ICU, intensive care unit

According to our lean model of Six Sigma quality improvement (QI) principles, to achieve optimal results, hospitals must maintain and enhance their performance [8]. In a 15-bed ICU in southwest Minnesota, we have seen a spike in the utilization of central lines and an increase in CLABSI rates during the peaks of the pandemic. As illustrated in Figure 1, our device utilization rate (DUR) has been on a steady upward trajectory since the summer of 2021, reaching the highest rate of 64% in the months of October and November. Also noted in Figure 1 is the CLABSI rate, which is at its highest, 11.36%, coinciding with the peak of our DUR.

**Figure 2:**
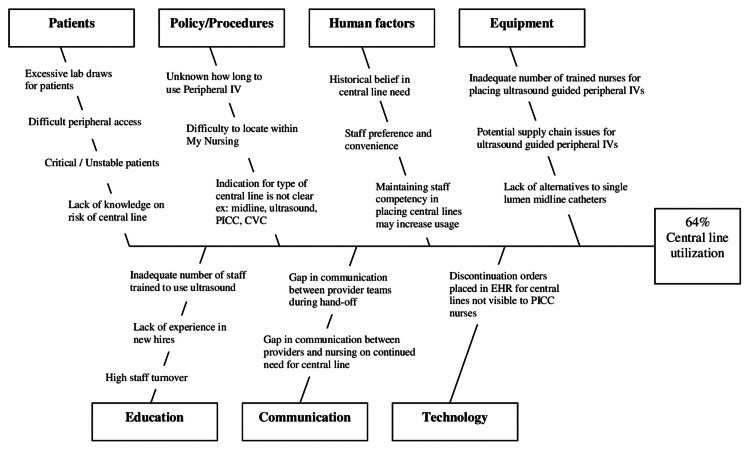
Fishbone diagram IV, intravenous; PICC, peripherally inserted central catheter; CVC, central venous catheterization; EHR, electronic health record

In January and February of 2022, we started this project's "socialization" and "discovery" phases. During this time, we had discussions about the increased DUR and CLABSI rate and the establishment of a multidisciplinary team that conducted a review of current practices and potential contributors to the increased DUR. The team (consisting of physicians, nurses, and research trainees) utilized the define, measure, analyze, improve, and control (DMAIC) model of Lean Six Sigma and the elements of the Standards for Quality Improvement Reporting Excellence (SQUIRE) guidelines [9]. This project aimed to decrease the DUR by 37.5% to reach our target DUR of 40% without adversely affecting staff satisfaction. The target population was all adult ICU patients. The potential contributors to an increased DUR were presented in the form of a fishbone diagram (Figure 2). The root causes of increased central line usage were determined to be a lack of awareness of appropriate central line indication and gaps in communication.

Study of the intervention and measures

To monitor our efforts to reduce central line utilization, device data was sourced from the electronic health record. Prior to the initiation of this project, survey data was collected from the 51 ICU staff members (consisting of physicians and nurses). The survey gauged staff satisfaction on a four-point scale. The results of this survey were compared and contrasted to the same survey that was administered at the end of the project to ascertain whether any changes had occurred during the duration of this project.

Ethical considerations

Institutional review board approval was not required. This QI study was granted ethics exemption by research ethics board. There were no conflicts of interest among the stakeholders. The project was funded internally without any commercial funding.

## Results

The total number of patients for the duration of the QI project included 119 adult patients, with 58% male, varied distribution of age, and the majority being White (92%) and English speakers (98%) (Table 1).

**Table 1:** Patients demographics **Others include Asian, Black or African American, Pacific Islander, Puerto Rican, Mexican, Hispanic, Latino, and choose not to disclose

Total Patients	Gender		Age Profile								Preferred		Race/Ethnicity	
	Male	Female	19-30	31-40	41-50	51-60	61-70	71-80	81-90	91+	English	Spanish	White	Others**
119	69	50	13	10	11	13	26	28	16	2	116	3	110	9

Improvement measure and countermeasure

**Figure 3:**
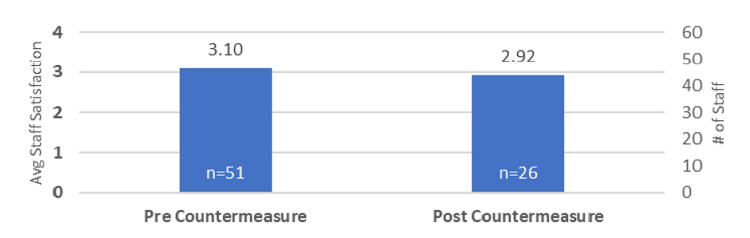
Countermeasure Avg: average

The numeric baseline for DUR (our improvement measure) was 64%. To calculate this, data was collected from October 1, 2021, to November 30, 2021. The numeric baseline of the countermeasure (staff satisfaction survey) was 3.1. Data for this countermeasure was collected from July 11, 2022, to July 25, 2022. When the countermeasure was reassessed at the end of the project, the numeric baseline was 2.92, as in Figure 3.

Intervention

Interventions were identified and implemented to address the critical barriers to decreasing utilization. The identified themes were process, communication, education, and closed-loop feedback.

Process

**Figure 4:**
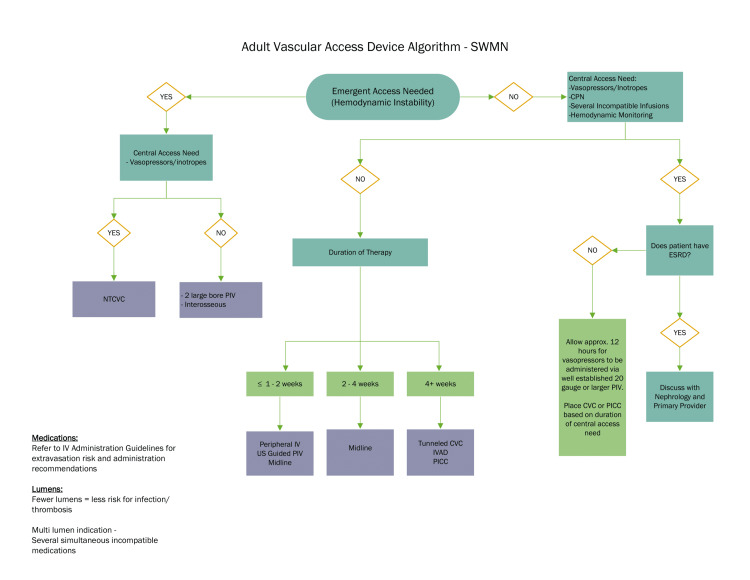
Adult vascular access device algorithm NTCVC, non-tunneled central venous catheter; PIV, peripheral intravenous; PICC, peripherally inserted central catheter; IVAD, implanted vascular access device; ESRD, end-stage renal disease; IV, intravenous; US, ultrasound; CVC, central venous catheterization; SWMN, southwest Minnesota; CPN, central parenteral nutrition

A vascular access algorithm was created to suggest the type of access needed based on the duration of the line and medication infusions (Figure 4). A rounding checklist included indications for central line placement and alternatives for vascular access.

Communication

Daily interdisciplinary rounds were enhanced, and evening rounds were implemented to discuss central line indications and barriers to the removal of the central line.

Education

Central line education was provided during nursing shift huddles and weekly emails. An educational poster was created and displayed in the ICU.

Closed-Loop Feedback

The result of the post-intervention numeric improvement measure and the sample size were tracked and recorded. Monthly data was compiled and distributed for review and discussed with the project group, and project milestones were recorded.

Average length of stay (ALOS)

The average length of stay (ALOS) at the beginning of the QI project was 3.15, and at the end of the project, the ALOS was lower at 2.45.

## Discussion

During the COVID pandemic, there was an increase in CLABSI in the ICU. Guidelines for adequate placement and maintenance of central line were being followed along with other measures to prevent CLABSI. Because of the concomitant increase in central line utilization, we designed this quality improvement project to decrease the DUR. We acknowledge that decreasing the DUR in an ICU may be challenging because of the acuity of illness seen in this population of patients; however, with education and twice-daily unit rounds, we were able to decrease the use of CVC, as well as earlier discontinuation of lines. The project brought awareness to the increased utilization of central lines. Discussions of the appropriate placement and early removal of central lines helped reduce our DUR. By June 2022, our DUR had decreased to less than 40% (Figure 1). The decrease in central line use in our ICU was completed through a defined process that included several patient care team members. The team continued to review utilization rates to ensure the sustainment of our goal. The staff satisfaction survey dropped by 0.18 points when we surveyed the staff again, possibly due to a decrease in respondents from 51 in the beginning to only 26 respondents in the final survey. We also observed a drop in the ALOS of patients in the ICU from an initial ALOS of 3.15 to 2.45 at the conclusion of the project.

This multidisciplinary QI study demonstrates the effective use of the define, measure, analyze, improve, and control (DMAIC) framework to improve patient safety and satisfaction by decreasing the use of central lines [10,11]. During the initial phases of this QI study, it was determined that our ICU had increased central line catheter utilization and subsequently increased rates of CLABSI. This is significant as it has been shown that the increased use of central lines is associated with CLABSI, which is linked with an increased risk of death, particularly among ICU patients [12]. In addition to infection and poor outcomes, central lines have been linked to increased discomfort and patient dissatisfaction [13,14]. Throughout the process of developing this project, it was found that a knowledge gap existed among the nursing staff and providers regarding the indications for the placement of central lines. To address this knowledge gap, the nursing staff utilized daily huddles to discuss central line indications and maintenance. Additionally, an educational poster was created and displayed within the ICU. Opportunities to increase the frequency of re-evaluation for the ongoing use of central lines and the potential for early discontinuation were addressed by the addition of evening rounds with providers and the nursing staff. Following these improvements in communication gaps and education on indications for central line placement, the utilization of central lines within the ICU was significantly decreased. To ensure that the efforts of this project are sustained, the DUR is reviewed monthly by the clinical nurse specialist and escalated to the ICU division, peripherally inserted central catheter (PICC) nurses, and charge nurses as needed. The impact of reducing the placement and duration of central lines will be improvements in patient outcomes, satisfaction, and experience [15].

CLABSI is a dreaded complication that we should avoid at all costs. Healthcare facilities have implemented bundles for the placement and maintenance of CVC to prevent CLABSI; however, despite these bundles, CLABSI continues to occur. Although these bundles include the removal of unnecessary lines in critical care units, this may be difficult to accomplish. The duration of line placement has been associated with an increased risk of CLABSI [16]. A prospective study from Malaysia found that the duration of device placement was a predictor of the development of bacteremia [17]. Another large study from Germany recommended minimizing catheter days [18].

Avoiding unnecessary CVC placement and the continued assessment of line needs is an important component of the bundles to prevent line infections. In our institution, many emphases were placed on aseptic technique of line and adequate maintenance, but despite this, we saw an increase in CLABSI cases. With the engagement of ICU staff, education, twice-daily assessment of the need for CVC, and the use of alternative line access such as ultrasound-guided peripherally intravenous, we were able to decrease the DUR with a subsequent decrease in CLABSI.

Limitations

There are some important limitations to consider in this quality improvement project. We implemented many strategies simultaneously to lower our DUR, including creating a rounding checklist, an educational poster, daily huddles to review indications, and a central line indication algorithm. It is difficult to identify which strategy was the most effective in reducing the DUR and eliminating CLABSI. The COVID pandemic was an important factor resulting in decreased DUR with a decrease in cases of COVID during the project implementation phase.

## Conclusions

Improvement in CVC utilization rate by closely monitoring the clinical need for line placement can lead to a decrease in CLABSI in critically ill patients and should not be overlooked in the bundles of CVC placement for the prevention of CLABSI. By ensuring that CVCs are only inserted when necessary, healthcare providers can effectively decrease the occurrence of CLABSI, benefiting the overall well-being of critically ill patients.
